# The Dark Side of Dendritic Cells: Development and Exploitation of Tolerogenic Activity That Favor Tumor Outgrowth and Immune Escape

**DOI:** 10.3389/fimmu.2013.00419

**Published:** 2013-12-02

**Authors:** Barbara Seliger, Chiara Massa

**Affiliations:** ^1^Institute for Medical Immunology, Martin Luther University Halle-Wittenberg, Halle (Saale), Germany

**Keywords:** dendritic cell, tumor, immune escape, tolerance, immunotherapy

## Abstract

Dendritic cells (DC) play a central role in the regulation of the immune responses by providing the information needed to decide between tolerance, ignorance, or active responses. For this reason different therapies aim at manipulating DC to obtain the desired response, such as enhanced cell-mediated toxicity against tumor and infected cells or the induction of tolerance in autoimmunity and transplantation. In the last decade studies performed in these settings have started to identify (some) molecules/factors involved in the acquisition of a tolerogenic DC phenotype as well as the underlying mechanisms of their regulatory function on different immune cell populations.

## Introduction

The immune system evolved with the difficult task of preserving the integrity of the “self,” while protecting it from “non-self” and/or dangerous invaders, thus finding the right balance between aggression and tolerance. A central role in orchestrating the different immune cell subpopulations is played by dendritic cells (DC), the major professional antigen presenting cells (APC). Over the last two decades, many different DC subsets have been identified and classified into myeloid DC, which comprise all monocyte-derived cells and blood-resident CD1c^+^ DC and into plasmacytoid DC (PDC).

A particularly difficult task for the immune system is to fight tumors, since they derive from the “self,” but based on their high proliferative potential they are dangerous for the survival of the host. Moreover, due to the high mutation rates of tumor cells the selection pressure posed by an immune response can result in tumor immunoediting with the outgrowth of immune escape variants or the induction of a suppressive microenvironment. In line with the central role of DC in balancing response versus tolerance, many of the immune escape mechanisms displayed by cancer cells affect DC. These include alterations in the frequency and/or function of circulating and tumor-infiltrating DC in patients with tumors of different histologies. In particular, DC in cancer patients can be affected in their differentiation capacity, with either enhanced apoptosis or skewed phenotype toward immature cells with suppressive properties collectively named myeloid derived suppressor cells [MDSC; (1, 2)], in their ability to process and/or present tumor-associated antigens (TAA) and in their ability to interact with effector cells, e.g., to activate and/or correctly polarize them.

Studies performed with DC differentiated *in vitro* in the presence of tumor cells or of their conditioned medium as well as with purified tumor-infiltrating DC identified the underlying mechanisms responsible for such alterations leading to a pro-tumorigenic phenotype. This review summarizes the known processes employed by tumor cells to subvert professional APC (summarized in Table 1) and how the increasing knowledge can not only help in fighting cancer, but also in inducing tolerance to transplanted organs and suppression of autoimmune diseases.

**Table 1:** **Effects of tumor-derived molecules on APC functions**.

APC properties	Factor	Effects^a^	Reference
Differentiation	Ganglioside	Reduced CD1a. Reduced DC from CD34 progenitor	(77, 78)
	HA	Suppressive Mf promoted over DC	(18)
	HLA-G	Promoted expansion of MDSC *in vivo*	(27)
	Lactate/pH	Impaired differentiation (no CD1a), promoted MDSC expansion	(4, 6)
	Mucins	More immature phenotype	(59, 60)
	PGE_2_	Promoted MDSC differentiation	(69)
	VEGF	Promoted MDSC differentiation	(40 –42)
	Wnt5a	Impaired differentiation of monocytes toward mDC	(53, 55)
Migration	Ganglioside	Lower CCR7 and impaired migration toward CCL19 (LC) and CCL3	(74, 78)
	Hypoxia	Enhanced migration toward SDF-1α and CCL4; reduced CCR7 levels	(8, 9)
	PGE_2_	Enhanced expression and functionality of CCR7 (mDC). Reduced CCR7/CXCR4 ratio for tissue retention (PDC)	(62, 100)
	TGF-β	Reduced migration *in vivo* to LN and *in vitro* to CCL19; enhanced expression of inflammatory CCR	(44, 45)
Ag uptake and processing	Ganglioside	Reduced expression of various APM components; reduced endocytosis	(78, 79)
	HLA-G	Reduced MHC class II antigen processing	(22)
	Hypoxia	Reduced endocytosis	(8, 9)
	TGF-β	Reduced endocytosis and phagocytosis	(44)
	Wnt5a	Lower fluid phase and CD206-mediated Ag internalization	(55)
Surface molecules	Ganglioside	Lower CD40, CD54, CD80, CD86, CD83 (LC, mDC)	(74, 77, 82)
	Glycodelin	Reduced CD83 and CD86	(33)
	HLA-G	Reduced HLA-DR, CD80 and CD86	(22, 24 –26)
	Hypoxia	Reduced CD40 and HLA-DR	(8)
	IL-10	Reduced CD86	(177)
	Mucin	Reduced CD40, CD83 and CD86	(58 –60)
	PGE_2_	Enhanced OX40L and CD70 induction (mDC). Reduced CD40 (PDC)	(63, 64, 100)
	TGF-β	Reduced CD80 and CD40	(44)
	Wnt5a	Reduced CD80 and CD86 (PDC)	(56)
Secreted molecules	Ganglioside	Reduced IL-6, IL-12 and TNF-α, increased PGE_2_ secretion	(59, 82)
	Glycodelin	Enhanced IL-6 by monocytes and Mf. Reduced IL-12 and higher IL-10 in mDC	(33, 34)
	HA	Enhanced IL-10 by suppressive Mf. Reduced IL-12/IL-10 ratio in mDC	(18, 19)
	HLA-G	Reduced IL-12, enhanced IL-6	(24, 26)
	Hypoxia	Reduced IL-12 and TNF-α and enhanced IL-10	(8)
	IL-10	Reduced IL-12 and/or IFN-α production (PDC)	(101, 102)
	Lactate/pH	Reduced IL-12, IL-6 and TNF-α; enhanced IL23	(5, 6)
	Mucin	Reduced IL-12, increased IL-10	(58 –60)
	PGE_2_	Reduced IL-12/IL23 ratio, reduced CXCL10, CCL5 and CCL19; enhanced IDO (mDC). Reduced IFN-α and TNF-α (PDC)	(66, 70, 71, 100, 102)
	sCD83	Enhanced TGF-β and consequently IDO (PDC, mDC)	(51)
	TGF-β	Reduced IFN-α and TNF-α (PDC)	(100, 103)
	Wnt5a	Inhibited IFN-α secretion (PDC); enhanced TGF-β and IL-10; reduced IL6 and IL-12	(54–56)
Survival	Ganglioside	Enhanced apoptosis (LC, mDC)	(74, 75)
	Glycodelin	Contradictory results	(34)
	HA	Enhanced apoptosis via NO induction	(20)
	IL-10	Enhanced apoptosis (PDC)	(101)
	Mucins	Enhanced apoptosis early during differentiation	(57)
Interaction with NK cells	HLA-G	Reduced activation (CD69, IFN-γ secretion, cytotoxicity)	(26)
	PGE_2_	Reduced recruitment and induction of IFN-γ	(71)
Interaction with nk-T cells	TGF-β	Reduced CD1d and lipid presentation	(46)
Interaction with T cells	Ganglioside	Reduced allo-MLR (LC). Reduced proliferation to TT and allo-MLR (mDC)	(74, 78, 81, 82)
	Glycodelin	Reduced induction of proliferation. Reduced IFN-γ secretion	(28, 34)
	HA	Enhanced T cell apoptosis via ROS production	(19)
	HLA-G	Reduced allo-MLR, more IL-10 secreting CD8^+^ T, anergic CD4^+^ T	(22, 25)
	Hypoxia	Enhanced IL-4 over IFN-γ secretion, type 2 skew	(8)
	IL-10	Enhanced proliferation of CD4^+^ T and skew toward Th2 (PDC). Reduced allo-MLR and anergy induction	(102, 177)
	Lactate/pH	Reduced Ag specific CD8^+^ T proliferation; enhanced IL-17 over IFN-γ secretion	(5, 6)
	Mucin	Reduced allo-MLR, reduced IFN-γ secretion by CD8^+^ T	(60)
	PGE_2_	Enhanced IL-17 and reduced IFN-γ, inhibition via IDO and soluble CD25 (mDC). Enhanced proliferation of CD4^+^ T and skew toward Th2 (PDC)	(65, 66, 70, 102)
	sCD83	Induction/expansion of CD4^+^ CD25^+^ Foxp3^+^ Treg	(51)
	TGF-β	Reduced proliferation in allo-MLR and to peptide, reduced IFN-γ secretion	(44)
	Wnt5a	Reduced IFN-γ secretion, higher IL-10 secretion. Reduced proliferation	(54, 55)

*^a^ When nothing given between brackets, mDC are considered*.

*PDC, plasmacytoid DC; LC, Langerhans cells; Mf, macrophages; mDC, myeloid DC; LN, lymph node; MLR, mixed leukocyte reaction*.

## Myeloid DC and Cancer

Tumor cells can influence the phenotype and function of myeloid cells at different time points of their life and with distinct mechanisms. These include the metabolic shift of tumor cells toward the anaerobic glycolytic pathway for glucose degradation resulting in increased concentrations of extracellular lactate and an acidification of the microenvironment, the so-called Warburg effect (3). Monocytes cultured *in vitro* in the presence of lactate and low pH have shown an impaired differentiation toward DC favoring either an expansion of MDSC (4) or of macrophages that promote a Th17 polarization (5). Despite prolonged incubation in the presence of lactate impairs DC responsiveness to lipopolysaccharide [LPS; (6)], a transient exposition promotes DC maturation and enhances their ability to induce a type 1 immune response (7). In addition to pH alterations, the tumor microenvironment is characterized by hypoxia that skews DC toward a type 2 polarization (8), reduces their ability to uptake antigens (Ag), and alters their migratory properties (9).

In addition, expression of hyaluronan (HA), a component of the extracellular matrix of the tumor stroma, correlates with tumor invasiveness and poor survival of patients with ovarian, breast, and colorectal cancer (10–13), while high HA levels correlate with more differentiated tumor phenotype and an enhanced survival in patients with oral squamous carcinoma (14). The effects of HA on DC are controversial and possibly related to its size: whereas low molecular weight HA can induce DC maturation *in vitro* (15, 16) and improve their functionality *in vivo* as cancer vaccine (17), intermediate sized HA impairs monocyte differentiation resulting in immunosuppressive APC characterized by a macrophage-like phenotype (CD14^+^, CD1a^low^), a reduced upregulation of costimulatory molecules and inflammatory cytokines after stimulation with toll-like receptor (TLR) ligands and an enhanced secretion of interleukin (IL)-10 (18, 19). Moreover, HA-conditioned DC can secrete nitric oxide (NO) and reactive oxygen species (ROS) that can induce apoptosis in DC and in co-cultured T cells, respectively (19, 20).

An other escape strategy exploited by tumor cells is the hijacking of endogenous mechanisms of tolerance induction used by immuno-privileged organs. This is mediated by the non-classical HLA-G antigen, which exhibit a tightly controlled physiologic expression restricted to cornea, thymic epithelial cells, reproductive organs, embryonal tissues, and the extravillous cytotrophoblasts at the maternal-fetal interface. Furthermore, HLA-G is often expressed in solid and hematologic tumors either as a transmembrane and/or a secreted/shed protein, thereby protecting tumor cells from the cytolytic activity of natural killer (NK) and T cells (21). In addition, HLA-G can also impair myeloid DC by binding to the inhibitory receptors ILT2 and ILT4 in humans and PIR-B in mice (22–24). Receptor triggering by HLA-G inhibits the nuclear translocation of the transcription factor NF-κB (25), which is consequently accompanied by reduced expression of costimulatory molecules and proinflammatory cytokines as well as impaired presentation of MHC class II-restricted epitopes (22). As a consequence, HLA-G treated DC lack the ability to induce NK cells activation (26) and promote anergy of effector cells and differentiation of regulatory T cell [Treg; (22)]. Furthermore, tumor-expressed HLA-G induced suppressive MDSC and tumor growth *in vivo* (27).

Glycodelin (previously called placental protein 14 or PP14, α2-globulin, progesterone-associated endometrial protein or zona-binding inhibitory factor) has been originally identified as the molecule responsible for the immunosuppressive activity in the decidua during early gestation (28), but is also expressed in tumors of the reproductive tract, e.g., ovarian carcinoma, where its glycosylated form glycodelin A (GdA) correlated with unfavorable prognosis (29). Furthermore, glycodelin correlate with a worse patients’ prognosis in familiar, non-BRCA1/2 breast carcinoma (30) and in lung cancer (31). *In vitro* characterization of glycodelin function demonstrated suppressive effects on all immune cell populations (32), including DC. Treatment of DC with GdA results in lower expression levels of costimulatory molecules, a low IL-12/IL-10 ratio (33), and a reduced ability to induce a type 1 polarization of effector cells (34). Depending on the culture conditions, GdA has also been reported to induce or suppress apoptosis in monocytes [see discussion in Ref. (34)].

Other “physiologic” tolerogenic factors borrowed by tumor cells include indoleamine 2,3-dioxygenase [IDO; (35)], adenosine production via CD73 expression (36), and secretion of IL-10 (37, 38), transforming growth factor-β [TGF-β; (39)] or soluble CD83 (sCD83).

Transforming growth factor-β plays not only a role in MDSC development, like vascular endothelial growth factor [VEGF; (40–42)], IL-6 and/or macrophage colony stimulating factor [M-CSF; (43)], but also impairs the DC migratory capacity by altering the expression pattern of chemokine receptors (44, 45) and inducing downregulation of CD1d thus impairing DC interactions with NK-T cells (46).

sCD83 was found in total blood cell cultures after stimulation and might represent a feed back mechanism to shut down an immune response (47). Indeed, enhanced serum levels of sCD83 detected in hematologic malignancies and solid tumors, like lung carcinoma correlate with shorter tumor-free survival (48–50). *In vitro* treatment of DC with recombinant sCD83 results in enhanced IDO production and induction of TGF-β producing Foxp3^+^ Treg (51).

An additional strategy of immune escape mechanisms exploited by tumor cells consists in the upregulation of molecules with negative effects on DC. These include alterations in the Wnt/β-catenin pathway inducing activation of MerTK receptor (c-mer proto-oncogene tyrosine kinase) in infiltrating cells like macrophages and DC that help tumor growth *in vivo* (52). *In vitro* studies have found that tumor-derived Wnt5a can impair the differentiation of monocytes toward DC (53) and inhibit the maturation response to TLR ligand by myeloid DC (54, 55) as well as by PDC (56).

Mucins are expressed by many epithelial tumors and their presence during differentiation of monocytes toward DC results in less differentiated cells with increased apoptosis (57), impaired response to TLR ligand stimulation, cytokine production skewed toward the immunosuppressive IL-10, impaired ability to induce proliferation of T cells, and enhanced induction of suppressive T cells (58–60). Those effects seem to be mediated by binding to the mannose receptor, siglec-3 and -9 (57–59).

A hallmark of many tumors is the secretion of high levels of prostaglandin E_2_ (PGE_2_) due to upregulation of cyclooxygenase (COX)1/2. The consequences of PGE_2_ on DC functionality are complex. While it represent a component of the “gold standard” cocktail for vaccine DC maturation (61) due to its role in promoting CCR7-mediated migration (62), and it also induces the expression of costimulatory molecules like OX40-L and CD70 promoting T cell functions (63, 64), it can inhibit the synthesis of IL-12p70 (65), while favoring the secretion of IL-23 that promote Th17 immune responses (66) and tumor development (67, 68). Moreover, PGE_2_ enhances MDSC differentiation (69), induces expression of IDO and soluble CD25 that inhibit T cell stimulation (70) and impairs the cross talk with NK cells (71). A possible explanation for the contrasting effects can be due to the specific receptor triggered by PGE_2_ (72) and/or the relative ratio between PGE_2_-treated DC and effector cells (73).

Altered and/or secreted gangliosides have also been demonstrated to affect DC differentiation and survival (74–78). Moreover, gangliosides impair the ability of monocytes to induce T cell proliferation due to a downregulated expression of components of the antigen processing machinery [APM; (79, 80)], a suppressed costimulation and a reduced cytokine production (81, 82). *In vivo*, a correlation between elevated levels of the ganglioside GM3 and a higher frequency of immature DC was found in non-small-cell lung cancer (83).

Furthermore, “tumor-deviated” DC/MDSC exhibit an altered phosphorylation pattern of STAT3 (84, 85) that has also been linked to the inhibition of IL-12p40 transcription (86) and/or of p38 (87) that is involved in the induction of Th17 responses (88).

In addition to boost the immune suppression, tumor-conditioned DC can also provide direct help to tumor cells by secreting mitogens for the tumor cells (89), by favoring the epithelial mesenchymal transition (90), by promoting their invasiveness and ability to metastasize (8, 90) and by inducing angiogenesis (91, 92).

## PDC and Cancer

Plasmacytoid DC have been found in the infiltrate of various human solid tumors like melanoma, breast, ovarian, and head and neck carcinoma, where they frequently correlated with a worse patients’ prognosis (93–96). Functionally, PDC can be recruited by the tumor through its secretion of CXCL12 (also called SDF-1α) and CCL20 (97–99). Then, factors locally released by tumor cells, like TGF-β, tumor necrosis factor (TNF)-α, IL-10, and PEG_2_ (95, 100–104) as well as triggering of the PDC-specific receptor ILT7 (105, 106) induce the immunosuppressive properties of PDC. Indeed, tumor-conditioned PDC display a semi-mature phenotype with expression of costimulatory molecules but impaired secretion of IFN-α (93, 101, 103). In addition, tumor-associated, tolerogenic PDC showed an upregulation of the transcription factor Foxo3 (107) and an impaired migration to lymphoid organs due to reduced CCR7 expression (100).

Characterization of the immunosuppressive activity of PDC *in vitro* have highlighted their ability to induce unresponsiveness of effector cells, to promote the development of suppressive CD8^+^ T cells, to differentiate naïve CD4^+^ T cells toward Foxp3^+^ or IL-10 producing Treg as well as to expand pre-existing Treg (108–113). From the molecular point of view, important roles have been identified for ICOS ligand (ICOS-L), IDO, notch ligand delta-like 4 (Dll4), and granzyme B. ICOS-L is upregulated shortly after maturation induced by CD40-L or TLR9 triggering (108), is involved in inducing IL-10 production in CD45RO^+^ T cells (114) and in sustaining the survival and proliferation of Foxp3^+^ Treg (115). A role *in vivo* for this pathway is supported by the co-localization between ICOS^+^ Treg and ICOS-L^+^ PDC within breast and ovarian carcinoma (115, 116). Murine and human PDC can produce IDO *in vitro* upon triggering of TLR9, CTLA-4, GITR, or CD200 (117–119). PDC expressing IDO have been identified in melanoma draining lymph nodes in murine models and human patients and have been correlated with the activation of naïve and mature Treg (120–122). In murine models, the constitutively expressed Dll4 allow PDC to induce Th1 cells to produce IL-10 even under type 1 polarizing conditions, thus favoring the shut down of an immune response (123). Granzyme B, whose secretion by PDC is boosted by tumor-derived IL-3 and IL-10, is involved in the downregulation of the CD3ζ chain of T effector cells, thereby resulting in their anergy or deletion by apoptosis induction (124, 125).

In addition to their immunosuppressive role, PDC play a pro-tumorigenic role by promoting angiogenesis via secretion of TNF-α and IL-8 (126) and favoring metastasis dissemination into the bone (127).

## Improved Protocols for DC-Based Vaccination Against Cancer

**Figure 1:**
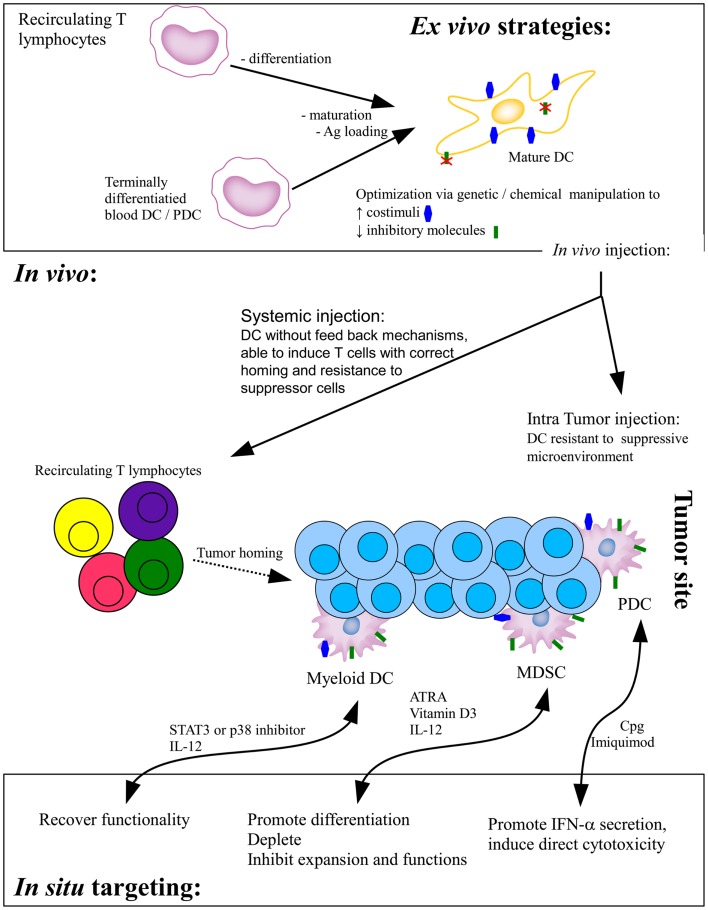
**Current DC-based strategies of tumor immunotherapy**. In the *ex vivo* strategy, monocytes-derived immature DC or terminally differentiated blood DC are loaded with tumor antigens and/or induced to mature before *in vivo* injections. Whereas systemically injected DC will migrate to the draining lymph node to prime effector T cells, intratumorally injected DC have to interact with effector cells within the suppressed microenvironment. Direct *in situ* targeting strategies aim at recovering the functionality of infiltrating DC, either promoting their correct differentiation or providing stimuli to foster their functionality. Ag, antigen; ATRA, all-trans retinoic acid.

Two major strategies of DC-based tumor immunotherapy have been implemented. The first is based on the *ex vivo* production and manipulation of DC that are then injected into the patients while the second targets the DC directly *in vivo* (Figure 1).

The classical strategy for the first approach consists in the differentiation of CD14^+^ circulating monocytes or CD34^+^-mobilized precursor cells into immature DC by culturing them in the presence of granulocyte-monocyte colony stimulating factor (GM-CSF) and IL-4 for 7 days, after which they are loaded with TAA and induced to mature before *in vivo* injection. Studies performed using cells from patients with different solid as well as hematologic cancer histotypes have demonstrated that precursor cells are either not irreversibly impaired and can be matured with this protocol or that is possible to rescue their differentiation into functional DC upon inhibition of STAT3, p38, and/or IL-6 (128, 129). Despite the good results obtained *in vitro* with patient-derived DC, and the induction of immune response in treated patients demonstrated by expanded Ag-specific T cells and delayed type hypersensitivity (DTH) reactions, the first clinical trials with vaccine DC resulted in poor clinical outcome. Based on the increased knowledge of the DC biology and of tumor escape mechanisms the protocol for the *ex vivo* production of vaccine DC has to be optimized (Box 1).

Box 1 **Optimization of DC-based tumor immunotherapy.**

Different anatomical and tumor derived factors pose problems to the success of DC-based therapy. Following is a list of key points that have to be optimized.

(A) *Ex vivo* DC preparation
  - DC subset: terminally differentiated blood DC (PDC, CD1c^+^ DC, mixed) or monocyte derived DC (GM-CSF + IL-4, GM-CSF + IFN-α, GM-CSF + IL-15; standard 7 days or shortened 2–3 days protocol).
  - Antigen loading: protein, DNA, or mRNA; one or multiple Ag, defined or total tumor repertoire.
  - Maturation: TLR-ligand(s) (poly IC, MPLA, R848) and/or immune-derived (CD40-L, IFN-γ). Is PGE2 to be added for the migratory ability?
  - Targeted effector cells: CD8^+^ T cell only; also CD4^+^ T helper and/or innate effector cells (NK, iNKT, γδ T cells)
(B) Vaccination protocol:
  - Injection route: intratumor versus systemic (intradermal, intramuscular, subcutaneous, intranodal)
  - Number of injection and distance in between
  - Optimal DC dose
  - Combination with other treatment modalities (remove suppressive populations, reduce local immunosuppression, enhance tumor permeability…)

The initial poor results of DC-based immunotherapy could be due to the immature or only partially mature phenotype of the DC, and in particular to their reduced levels of IL-12 secretion. Thus, many alternative maturation protocols have been developed, which induce DC with an enhanced IL-12 secretion and functionality *in vitro*, with some of them that have also reached clinical application. The “alpha type-1 polarized DC” obtained upon maturation in the presence of IL-1β, IFN-α, IFN-γ, poly IC, and TNF-α (130) have been tested in patients with recurrent glioma (131, 132), melanoma, and colorectal cancer (NCT00390338 and NCT00558051 at http://www.clinicaltrials.gov, respectively), whereas DC stimulated with LPS and IFN-γ have been used for the treatment of patients with breast cancer (133, 134). DC stimulated with the streptococcus-derived immunotherapeutic agent OK432 have been used against hepatocellular carcinoma (135) and colorectal cancer (136).

In parallel to the manipulation of the maturation protocol, the type of DC was also optimized. Alternative differentiation protocols for monocytes have been tested to obtain more physiologic DC types. GM-CSF has been combined with IFN-α to induce inflammatory DC, which have already been tested in patients with medullary thyroid carcinoma (137), or with IL-15 to induce Langerhans-like DC that despite the enhanced functionality *in vitro* did not provide higher responses in melanoma patients when compared to standard DC (138). Furthermore, terminally differentiated DC have been used both as single or mixed populations. Regarding the myeloid subset, in a preclinical trial sufficient amount of CD1c^+^ blood DC have been purified from healthy as well as melanoma and Bowel cancer disease patients under GMP (good manufacturing practice) conditions and could be induced to secrete proinflammatory cytokines, thus opening the way for a possible clinical application (139). Two different approaches using PDC have been developed. A leukemic cell line with PDC characteristic has been isolated and, after having demonstrated functional activity in humanized murine models (140) and with melanoma PBL *in vitro* (141) will be evaluated in a clinical trial in HLA-matched melanoma patients (NCT01863108). In contrast, de Vries and co-workers have employed autologous, patient-derived PDC in a phase I clinical trial against melanoma (142). A GMP platform has been established to purify all subtypes of circulating APC resulting in a population able to induce Ag-specific CTL both from healthy donors and myeloma patients (143). The injection of a highly purified DC population does not seem to be required since the sipuleucel-T (also called APC8015 or Provenge^®^) vaccination approved by the Food and Drug Administration (FDA) for treatment of prostate cancer patients is based on a highly mixed population, in which the DC targeted with TAA only represent a small component (144, 145).

Other optimizations have been also evaluated in order to modulate the suppressive environment that impair the *in vivo* ability of the vaccine DC to prime immune responses. This is mediated by rendering DC insensitive to the tumor-induced suppressive microenvironment by blocking inhibitory signaling pathways, like TGF-β (146, 147), IL-6 (148), and STAT3 (129, 149, 150). On the other side, the costimulatory function of DC have been further improved by providing the T lymphocytes with all required positive signals and/or the absence of negative feedback regulators in order to acquire full functionality and resistance to suppressor cells. DC unable to produce IL-10 (151, 152), insensitive to CTLA-4 triggering (153), providing enhanced levels of CD70 (154–157), CD80 (154), or GITR-L costimulation (153, 158) have proved to induce T cells with enhanced resistance to Treg suppression, delayed induction of tolerance as well as reversion of the tolerized status. Some of those “costimulatory enhanced” DC have also started the path of clinical trials like the TriMix DC (expressing CD40L, CD70, and a constitutively active TLR4 receptor) in melanoma patients (159, 160).

To provide a more general pro-stimulatory phenotype, multiple signaling pathways have also been enhanced by either inducing expression of the transcription factor T-bet (161, 162) or by silencing A20, an inhibitor of signaling pathway downstream of TLR and TNF receptors (163) resulting in DC with improved functionality. Similarly, with the increased knowledge of the important role of micro RNA (miR) in the fine tuning of gene transcription and their role in the immune response and in DC functions (164), DC-specific miR have been targeted. For example, inhibition of miR-22 and miR-503, two miRNA upregulated in DC upon coculture with tumor cells, resulted in improved therapeutic activity due to enhanced DC survival (165).

The major aim of the second line of therapy is to revert the tolerized phenotype of tumor-infiltrating and/or recirculating DC in order to allow proper activation of effector cells. The most clinically advanced strategies are those focusing on PDC and using ligands of the TLR-7 and -9 to recover their IFN-α secreting capabilities. After the successful use of imiquimod and CpG-containing oligonucleotides in murine models, many clinical trials have also been performed (166), leading to the approval of imiquimod for cancer immunotherapy by the European Medicines Agency and the FDA. A problem with such a strategy is the fact that in some, but not all tumors a downregulation of the two TLR in tumor-infiltrating PDC have been reported (93). At the basis of the positive outcome upon PDC *in situ* triggering there can be not only the activation of other immune cells and the inhibition of Treg (167), but also a direct tumoricidal activity upon upregulation of TRAIL and granzyme B (168, 169). Is to be underlined that the upregulation of granzyme B by PDC can also have detrimental effect by killing T cells (124, 125, 169). An other reported effect of CpG injection is the differentiation of MDSC toward functional monocytes with consequent reduction in the amount of suppressive cells (170, 171).

On the side of targeting myeloid DC *in vivo*, similar approaches to the *ex vivo* manipulation have been tested. Chemical inhibitors of negative signaling pathways like STAT3 (150), or positive modulators like miR-155 (172), have been injected *in vivo* with the aim to be uptaken by DC and to revert their tolerogenic phenotype and promote immune-mediated tumor rejection. Provision of missing IL-12 through different technical approaches have demonstrated that in addition to the stimulation of NK and T cells, also myeloid cells are positively affected with the activation of cytotoxic macrophages and reversion of MDSC with loss of their suppressive properties (173, 174). Many different strategies are also aiming at the removal of MDSC acting on their differentiation and/or suppressive functions (175). For example all-trans retinoic acid (ATRA), a compound reducing MDSC number and function has been combined to DC vaccination in a phase I trial in small cell lung cancer patients (176).

## Exploitation of Tolerogenic DC in Transplantation and Autoimmunity

In the setting of autoimmunity and transplantation the aim of immunotherapy is to reduce inflammation and to induce a local and/or antigen-specific immunosuppression/tolerance in order to avoid organ rejection and reduce the disease score without increasing the risk of opportunistic infections. Like for tumor immunotherapy, DC have been manipulated *ex vivo* or directly targeted *in situ*.

Tolerogenic DC have been differentiated *in vitro* from human monocytes and murine bone marrow cells upon culture in the presence of different combination of IL-10 (177), TGF-β (178), vitamin D3 (179), dexamethasone (180), protein kinase C inhibitor (181), and rapamycin (182). These cells are characterized by a semi-mature phenotype, the ability to expand Treg and to preserve such properties even in an inflamed microenvironment, as mimicked by stimulation with TLR ligands (183, 184). Murine models of organ transplantation as well as different autoimmune diseases have demonstrated the therapeutic applicability of such tolerogenic DC (185–188) and opened the way to preclinical evaluation in multiple sclerosis (189) as well as in a phase I trial in rheumatoid arthritis [RA; (190)]. Similarly, the good results obtained with non-obese diabetic (NOD) mice injected with DC silenced in the major costimulatory molecules [i.e., CD40, CD80 and CD86; (191)] have opened the way for a phase I safety study in patients with type 1 diabetes (192). In the setting of organ transplantation, “classical” murine bone marrow-derived DC from the organ donor have been either triggered with tetramer of sHLA-G1 (25) or silenced in NF-κB (193) in order to induce a transplant-specific tolerance that allow (longer) acceptance of the graft.

Direct *in situ* targeting of DC has also been implemented in order to promote local and/or antigen specific tolerance. Clinical trials have been performed with apilimod (or STA5326), a specific inhibitor of IL-12 and IL-23, which are the central mediators of the Th1 and Th17 responses involved in autoimmunity. Whereas in psoriasis a reduction in inflammatory cytokine and DC infiltration of the skin lesions was observed upon apilimod (194), no robust clinical improvement was found in RA (195) and contrasting results were reported in Crohn’s disease (196, 197). Although still in the preclinical phase systemic or topic injection of sCD83 was able to prolong survival of grafted organs (51, 198) as well as to reduce experimental autoimmune encephalomyelitis (EAE) in both prophylactic and therapeutic setting (199). Additional strategies inducing antigen specific tolerance consist in coupling the desired antigen to antibodies or ligands for specific receptors expressed by DC. Examples are DEC-205 (200, 201), the human DC immunoreceptor (DCIR) (202, 203), and the murine acid binding Ig-like lectin H [siglec-H; (204)] that induce specific tolerance to the antigen they have been targeted with.

## Conclusion

Despite the ever growing knowledge on the immunologic function of DC and how tumor cells try to subvert them, a long way has still to be performed before defining the best protocol of vaccination regarding not only the maturation/resistance of the DC but also the road of injection, the number of injections, the type of antigen(s) and the loading strategy (see Box 1). Of particular interest is the recent report that in therapeutic setting a single immunization performs better alone that with a following boost, a setting that is on the contrary highly favorable in prophylactic immunization (205).

## Conflict of Interest Statement

The authors declare that the research was conducted in the absence of any commercial or financial relationships that could be construed as a potential conflict of interest.
